# Heterogeneity of executive functions among comorbid neurodevelopmental disorders

**DOI:** 10.1038/srep36566

**Published:** 2016-11-09

**Authors:** Dina R. Dajani, Maria M. Llabre, Mary Beth Nebel, Stewart H. Mostofsky, Lucina Q. Uddin

**Affiliations:** 1 Department of Psychology, University of Miami, Coral Gables, FL, 33124, USA; 2 Center for Neurodevelopmental and Imaging Research, Kennedy Krieger Institute, 707 N. Broadway, Baltimore, MD, 21205, USA; 3 Department of Neurology, Johns Hopkins University School of Medicine, 733 N. Broadway, Baltimore, MD, 21205, USA; 4 Department of Psychiatry and Behavioral Sciences, Johns Hopkins University School of Medicine, 733 N. Broadway, Baltimore, MD, 21205, USA; 5 Neuroscience Program, University of Miami Miller School of Medicine, Miami, FL, 33136, USA

## Abstract

Executive functions (EFs) are used to set goals, plan for the future, inhibit maladaptive responses, and change behavior flexibly. Although some studies point to specific EF profiles in autism spectrum disorder (ASD) and attention-deficit/hyperactivity disorder (ADHD) — prevalent and often highly comorbid neurodevelopmental disorders — others have not differentiated them. The objective of the current study was to identify distinct profiles of EF across typically developing (TD) children and children with ASD and ADHD. We employed a latent profile analysis using indicators of EF (e.g., working memory, inhibition, and flexibility) in a mixed group of 8–13 year-olds including TD children (*n* = 128), children with ASD without ADHD (*n* = 30), children with ADHD (*n* = 93), and children with comorbid ASD and ADHD (*n* = 66). Three EF classes emerged: “above average,” “average,” and “impaired.” EF classes did not reproduce diagnostic categories, suggesting that differences in EF abilities are present within the ASD and ADHD groups. Further, greater EF dysfunction predicted more severe socioemotional problems, such as anxiety/depression. These results highlight the heterogeneity of current diagnostic groups and identify an “impaired” EF group, consisting of children with both ASD and ADHD, which could specifically be targeted for EF intervention.

Executive functions (EFs), the mental control processes needed to carry out goal-directed behaviors^1^, are fundamental to successful daily functioning across the lifespan^2^,^3^,^4^,^5^. EFs encompass several subdomains, including planning for future goals, inhibiting maladaptive responses, maintaining and manipulating information in working memory, and flexibly adapting behaviors to changes in the environment^6^. EFs are particularly important in children because EFs support the complex behaviors necessary for successful social interactions^7^. Impaired EFs are commonly observed in two prevalent neurodevelopmental disorders^8^: autism spectrum disorder (ASD, 1 in 68 children diagnosed with ASD^9^) and attention-deficit/hyperactivity disorder (ADHD, up to 1 in 15 children diagnosed with ADHD^10^). Given that EFs are malleable via intervention^11^, identifying EF deficits in childhood can facilitate improvements in EF with clinical intervention^12^. To identify children who would benefit from EF interventions, this study aimed to distinguish groups of children based on patterns of EF strengths and deficits, or “EF profiles.”

Although many studies have reported impaired EFs in ASD and ADHD^13^, there is considerable heterogeneity in the presentation of EF skills within these diagnostic groups. One previous study used community detection in a large sample based on various neuropsychological measures to identify subgroups of children with ADHD^14^. On the measures specific to EF (i.e., working memory and inhibition), an above-average and a below-average EF group emerged. To date, no formal subgroup analyses have been conducted in ASD based on EF. However, there is a multitude of conflicting evidence on EF dysfunction in ASD, pointing to heterogeneity in EF skills in this clinical population^15^,^16^. One consistent finding is more severe EF impairment in ASD than ADHD^8^,^13^,^17^,^18^. Heterogeneity within diagnostic categories may be resolved by identifying subgroups of children who have similar EF profiles.

There is mixed evidence concerning the consistency of EF impairment across subdomains of the construct (e.g., planning, inhibition, flexibility, working memory) in ASD and ADHD. A meta-analysis^19^ of performance-based measures of EF indicated that individuals with ADHD exhibited worse impairments in measures of response inhibition, vigilance, working memory, and planning than in flexibility. In ASD, most studies have focused specifically on planning and flexibility, revealing impairments in both subdomains^17^,^20^,^21^,^22^. Given these findings, it is unclear whether children with ASD and ADHD have either: (1) consistent EF abilities across subdomains, or (2) distinct EF profiles. To address this question, we used a latent profile analysis to identify patterns of strengths and deficits in EF subdomains.

While the Diagnostic and Statistical Manual (DSM-5^23^) considers ASD and ADHD to be distinct disorders, there is inadequate construct validity for these disorders based on EF^8^,^17^,^16^. The few studies that have examined specific EF profiles in ASD and ADHD employed variable-centered approaches^8^,^17^ (e.g., group averages), ignoring the heterogeneity within diagnostic categories. Van der Meer *et al.*^24^ accounted for this heterogeneity by employing a latent class analysis of cognitive and symptom measures in children with ASD and ADHD, and concluded that ASD and ADHD are part of one overarching disorder^24^. Further, there is considerable comorbidity of ADHD in ASD populations, ranging from 37–85%^25^. This heterogeneity and comorbidity between diagnostic groups hinders researchers aiming to identify effective treatments for these developmental disorders^26^.

As an alternative to *DSM*-based diagnoses, the scientific community is moving toward a neurobiological assessment of cognitive dysfunction, consistent with the Research Domain Criteria (RDoC) framework put forward by the National Institute of Mental Health^26^. The process of identifying homogenous subgroups of clinical populations could, in turn, lead to more targeted and effective treatments, which would propel the mental health field forward. EFs are a strong candidate to identify homogeneous subgroups because they are important for success throughout the lifespan and can be targeted for treatment and improved in children^11^,^27^,^28^. Intact EF is related to a wide range of behaviors that improve with training, such as social and academic abilities^28^,^29^. Therefore, identifying groups who have impaired EF can not only improve EF, but may also improve a wide range of critical daily-life functions.

Executive functions are notoriously difficult to measure^1^ due to factors such as their complexity and sensitivity to the context in which EFs are assessed^7^. Two common ways to measure EFs are performance-based measures, such as the classic Wisconsin Card Sorting Task^30^, and rating scales (completed by parents or teachers), such as the Behavior Rating Inventory of Executive Function (BRIEF^31^). Recent evidence suggests that performance-based and rating scales of EF capture different, but complementary, information^32^. Performance-based measures of EF tend to be highly structured, with the relevant goal provided by the experimenter, which may allow children to perform adequately during task administration in spite of executive dysfunction that would occur outside of the testing setting^18^. This is especially relevant when assessing children with ASD, who tend to perform better in structured than unstructured environments^33^,^34^. In a quantitative review by Toplak *et al.*^32^, the researchers posit that performance-based measures tap processing efficiency in the context of a structured environment, while rating scales capture success in rational goal pursuit in the context of unstructured environments. Because performance-based measures can capture process-specific information and rating scales can capture EF in complex, everyday environments, researchers have suggested using both types of measures to compose a “well rounded” understanding of children’s functioning^34^. With this in mind, we used rating scales supplemented by two performance-based measures of EF in the current study.

The first aim of this study was to delineate subgroups of children from a mixed group of typically developing children and children with ASD and ADHD based on patterns of EF strengths and deficits, or “EF profiles.” Prior research suggests that at least two EF subgroups are present within the ADHD population, including an at- or above-average group. Children with ASD may be characterized by more consistent and severe EF deficits than children with ADHD, which may constitute its own subgroup. Therefore, we hypothesized at least three distinct latent classes would emerge when examining a mixed group of TD, ASD, and ADHD children. The second aim of this study was to determine whether the EF classes would reproduce traditional diagnostic categories (TD, ASD, and ADHD). We hypothesized that the EF classes would consist of a mix of diagnostic groups. The third aim was to validate the clinical utility of these EF classes by relating class assignment with important socioemotional variables, such as anxiety/depression symptoms. Given that efficient EFs are related to a myriad of adaptive behaviors, we expected that greater EF impairment would be related to greater socioemotional problems.

## Methods

### Participants

Participants were 321 children (females: *n* = 69) ages 8–13 years (*M* = 10.01, *SD* = 1.27) with full-scale IQs ranging from 63 to 147 (*M* = 108.98, *SD* = 14.53) (Table 1). Participants were enrolled in one of two studies: one investigating motor skill learning in children with ASD and the other investigating motor physiology in children with ADHD. The sample contained a mixed group of typically developing (TD, *n* = 128) children, children with an ASD diagnosis without comorbid ADHD (*n* = 30), children with a primary ADHD diagnosis (*n* = 93), and children with ASD and comorbid ADHD (*n* = 66). There were 4 individuals with ASD who had missing comorbidity information. The majority of children with ADHD were diagnosed with the combined type (ADHD-C, 85%) and the remaining children with ADHD were the inattentive type (ADHD-I, 15%). TD children had no siblings with ASD and 11 TD children had a sibling with ADHD. Because we were not comparing diagnostic groups directly, we did not match groups on variables such as IQ. Written informed consent was obtained from all legal guardians and written assent was obtained from all children. All procedures were approved by the Institutional Review Board at the Johns Hopkins School of Medicine and all methods were carried out in accordance with the approved guidelines.

### Diagnostic Measures

The following measures were administered to confirm community diagnoses of autism: (1) the Autism Diagnostic Interview- Revised^35^ and (2) the Autism Diagnostic Observation Scale (the ADOS-Generic^36^ or the ADOS-2^37^, based on study enrollment date). To determine whether children with ASD had comorbid ADHD, they were also assessed with the Diagnostic Interview for Children and Adolescents, Fourth Edition (DICA-IV^38^). Community diagnoses of ADHD were confirmed with the: (1) DICA-IV^38^, (2) ADHD Rating Scale-IV, Home version (ADHD RS-IV^39^), and 3) the Conners’ Parent Rating Scales (the Revised, Long Version^40^ or the 3^rd^ Edition, Full-length^41^, based on study enrollment date). For more detailed exclusion and inclusion criteria see Supplemental Information online.

### Indicators of Executive Function for Latent Profile Analysis

Ten indicators of EF were used in the latent profile analysis, eight of which were subscales from a parent-report of EF (BRIEF^31^) and two of which were performance-based measures of EF (statue subtest of NEPSY-II^42^ and backward digit span of WISC-IV^43^). To take advantage of the complementary information parent-reports and performance-based measures provide, in addition to reducing measurement bias in the latent class variable, both parent-reports and performance-based measures were used.

#### BRIEF

The Behavior Rating Inventory of Executive Function (BRIEF^31^) is an informant-report of EF impairment of children 5–18 years of age. The parent-report form was used in this study and included the following subscales: inhibition, shift, emotional control, initiate, working memory, plan/organize, organization of materials, and monitor. The parent-report is reliable in normative (*r* = 0.81) and clinical samples (*r* = 0.79) and can distinguish clinical populations from TD children. The BRIEF has been shown to distinguish profiles of intact and impaired EF between children with ASD, ADHD-I, and ADHD-H^18^, making it an appropriate measure to determine EF profiles in this study. Higher scores indicate greater impairment, with T-scores ≥65 indicating clinical impairment. The T-scores (age- and gender-adjusted) were used for all of the subscales as indicators of the latent class variable.

#### NEPSY-II, statue subtest

The Developmental Neuropsychological Assessment (NEPSY-II^44^) is a battery of neuropsychological tests that include measures of EF. One such subtest, the statue, is a measure of children’s motor persistence and ability to inhibit responses to distracting stimuli. During the 75 s testing period, errors were recorded at 5 s intervals, including instances of talking, opening of the eyes, and body movements. Higher scores indicate better performance, with a maximum score of 30, indicating no errors were made. Although this task is designed for younger children, we used this measure since it had sufficient variability in our sample (range: 2–30, *M* = 23.68, *SD* = 7.08). This measure has been used in this sample of children in previous work^45^. The total score on the statue subtest was used as an indicator of the latent class variable.

#### WISC-IV, backward digit span

The Wechsler Intelligence Scale for Children IV (WISC-IV^43^) is a measure of intelligence for children ages 6–17 years. Full scale intelligence quotient (FSIQ) is an average measure of intelligence based on four indices: perceptual reasoning, verbal comprehension, processing speed, and working memory (WMI). One subtest of the WMI, the backward digit span (scaled at *M* = 10, *SD* = 3), is a measure of working memory maintenance and manipulation. Higher scores indicate greater ability, and scores of 8–12 are considered average. The backward digit span is internally consistent (*r* = 0.80) and reliable across time (*r* = 0.74)^46^. The scaled score of the backward digit span (age-adjusted) was used as an indicator of the latent class variable. We did not use the forward digit span in addition to the backward digit span: (1) to avoid having two highly correlated indicators in the model, and (2) because the backward digit span captures both maintenance and manipulation, whereas the forward digit span only measures maintenance.

### Distal Measures of Socioemotional Problems

#### CBCL

The Child Behavior Checklist^47^ is a parent-report of their child’s emotional and behavioral problems. There are nine syndrome scales that consist of problems that co-occur. Scales of interest were anxiety/depression, social problems, attention problems, and aggression. These scales are internally consistent (Cronbach’s α > 0.82), test-retest reliable (ICC = 0.95), and validly distinguish between children referred to clinics and nonreferred children^48^. Borderline clinical range for syndrome T-scores are ≥67; T-scores ≥70 are considered clinically elevated.

### Analytic approach

#### Latent Profile Analysis

**Figure 1:**
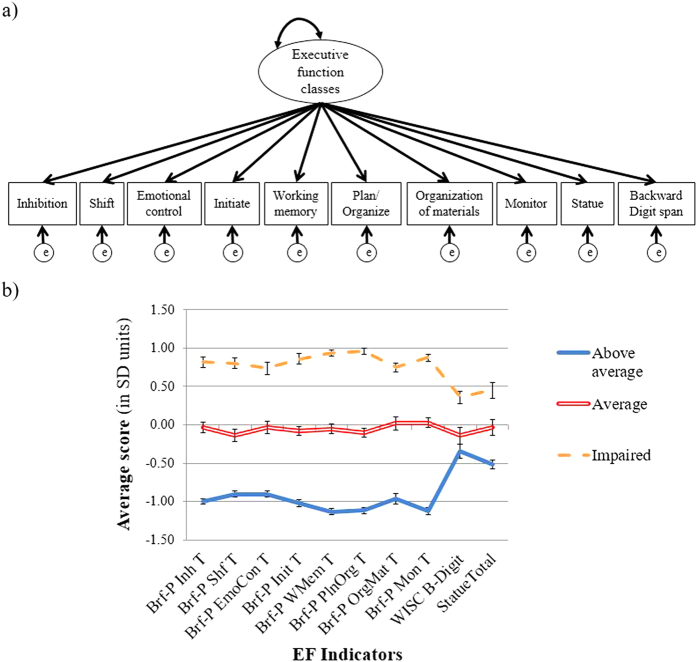
Latent Profile Analysis model and results. (**a**) The latent profile analysis had 10 indicators of executive function and (**b**) yielded three EF classes: “Above average”, “Average”, and “Impaired.” The three EF classes differed on their scores across the indicators. Scores are in standard deviation units based on data from the present sample. The WISC Backward Digit span and Statue Total were reverse-scored such that for all indicators, higher scores indicate greater impairment. Note for panel b: All EF indicators had a significant difference between Above average, Average, and Impaired groups except the WISC B-Digit, which only had a difference between Above average/Impaired groups and Average/Impaired groups. Brf-P Inh T: BRIEF-Parent report inhibition T-score; Shf T: Shift T-score; EmoConT: Emotional Control T-score; Init T: Initiate T-score; WMem T: Working memory T-score; PlnOrg T: Plan/Organize T-score; OrgMat: Organization of Materials T-score; Mon T: Monitor T-score; WISC B-Digit: Wechsler Intelligence Quotient for Children IV Backward Digit span scaled score (reverse scored); Statue Total: total score on the NEPSY-II statue subtest (reverse scored).

A latent profile analysis (LPA) was conducted using Mplus Version 7.4 to delineate subgroups of children who displayed differing patterns of EF strengths and deficits (Fig. 1a). Together, these indicators measure EFs such as inhibition, shifting, working memory, and planning/organizing. All EF indicators were normally distributed (absolute value of: skew < 1, kurtosis < 1.3).

Although we hypothesized at least three latent classes, no study to date has performed an LPA with a mixed clinical/TD group and this variety of indicators. Therefore, we followed an exploratory approach to identify the class number by testing increasingly more classes until the value of the log likelihood began to level off (1–6 latent classes). A significant value for the Lo, Mendell, Rubin Likelihood Ratio Test (LMR) indicates better model fit for the model with *k* classes compared to a model with *k-1* classes. The LMR was used to determine the maximum number of classes to consider, indicated by *k*-1 classes, given the model with k classes is the lowest class number with a non-significant LMR. Then, the following information criteria were used to decide between the remaining models: entropy, Akaike Information Criterion (AIC), Bayesian Information Criterion (BIC), and the sample-adjusted BIC (SA BIC). Higher entropy indicates better class separation. Lower values for AIC, BIC, and SA BIC indicate better model fit. Once the class number was chosen, a one-way analysis of variance (ANOVA) was conducted for each of the 10 indicators to characterize the differences in EF between classes. If a significant F test was obtained (*p* < 0.05), post-hoc analyses were conducted using Tukey’s HSD correction for multiple comparisons.

In an effort to include performance-based measures of EF in the latent profile analysis, the NEPSY statue subtest was included. However, the statue subtest may not be an ideal indicator for two reasons: (1) the raw score was not age-adjusted, and age is important to account for in studies of EF in children, and (2) this measure was not designed for use in children in the age range currently examined. Therefore, the latent profile analysis was repeated without using the NEPSY statue subtest to ensure this indicator did not have a significant effect on the formation of the classes. One participant was excluded from this analysis because they had missing data on all of the remaining 9 indicators.

#### Mixture Regression Analysis: Diagnosis

To determine whether the EF classes reproduced the diagnostic categories (TD, ASD, and ADHD), a mixture regression analysis was performed. To ensure that the structural model did not affect the assignment of classes in the measurement model, distal outcomes were treated as auxiliary variables in Mplus^49^. The Lanza method^50^ was used to estimate model parameters for the categorical distal outcome (diagnosis), due to its robustness against biased parameter estimations and the best preservation of the latent class variable compared to other methods (e.g., the 1-step approach)^49^. To calculate odds ratios using the Lanza method, the last EF class (the “average” EF class, see Results) was used as a reference, such that its odds ratio was always 1; ASD was used as the reference diagnostic group, and its odds ratio was always 1. Thus, not all odds ratios are interpretable, so the results of interest were the probabilities, interpretable odds ratios, and class difference tests (using the Wald χ^2^ test).

#### Mixture Regression Analysis: Socioemotional variables

To test whether the EF classes differed on behavioral measures of children’s functioning, additional mixture regression analyses were performed. A separate model was used for each of the four dependent (distal outcome) measures of socioemotional problems: anxiety/depression, social problems, attention problems, and aggressive behavior. Diagnosis was included as a categorical covariate. The distal outcomes were treated as auxiliary variables in Mplus using the manual 3-step method^49^. Significant differences in the continuous distal outcomes between classes were determined by non-overlapping 95% confidence intervals around the covariate-adjusted means of the distal outcome (the intercept). The mixture regression analyses were only completed for the 10-indicator model (including the NEPSY statue subtest).

## Results

### Latent Profile Analysis

According to the LMR, the maximum number of classes to consider was 3 (*p* = 0.07, Table 2). The three class model showed a relatively large decrement in the log likelihood value compared to the 2-class model, and the 3-class model had lower AIC, BIC, and SABIC values than the 2-class model. Furthermore, prior studies led us to believe that the minimum class number should be three^13^,^14^,^51^.

The first class (*N* = 105) had overall above average EFs (“above average”) (Fig. 1b, Supplementary Table S1). Most indicators were nearly a standard deviation below the sample’s mean (indicating better EF) on measures of the BRIEF. The “above average” class had the lowest (best) scores on the statue subtest compared with the other groups.

The second class (*N* = 78) had slightly below average scores on all of the EF indicators (“average”), with most scores on the BRIEF slightly higher than 50 but below the clinical cutoff of 65.

The third class (*N* = 138) had the poorest overall EF (“impaired”), with clinically elevated scores (BRIEF T-scores ≥ 65) on most measures. Exceptions were the emotional control and organizing materials subscales of the BRIEF, wherein the average score was slightly below 65 in the “impaired” class. The “impaired” class had the highest (poorest) statue subtest scores compared with the other EF classes.

Across all EF classes, the WISC-IV backward digit span was in the normal range. The “above average” and “average” classes did not differ on their backward digit span scores, but the “impaired” class performed significantly worse than both the “above average” and “average” classes on this measure. Overall, the “above average” class had the best performance on the EF indicators, followed by the “average” and “impaired” classes. The results of the latent profile analysis do not support the hypothesis that in a mixed group of children there are differences in patterns of EF strengths/deficits, but instead provide evidence that classes differ by their severity of EF dysfunction.

These results were largely replicated in the latent profile analysis excluding the NEPSY statue subtest, where the optimal class number was three and the classes primarily differed in their levels of EF, not in patterns of EF strengths/deficits (see Figure S1, Tables S4 and S5).

### Mixture Regression Analysis: Diagnosis

**Figure 2:**
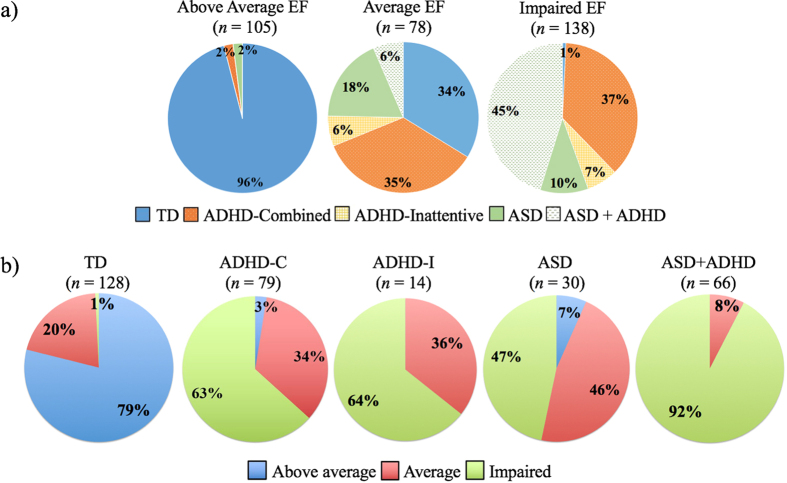
EF classes did not reproduce diagnostic groups. (**a**) Shown are the proportions of diagnostic groups within each EF class and (**b**) the proportions of the EF classes within each diagnostic group. ADHD-C: attention-deficit/hyperactivity disorder- combined type; ADHD-I: attention-deficit/hyperactivity disorder- inattentive type; ASD: autism spectrum disorder; ASD + ADHD: ASD with comorbid ADHD.

As hypothesized, these EF classes did not reproduce the groups based on clinical diagnosis (Fig. 2). The “above average” class was composed mainly of TD children, but the “average” and “impaired” classes contained a mix of diagnostic groups (Fig. 2a). Of note, two children with ADHD-C and two children with ASD were categorized into the “above average” class. The “average” class was composed of TD children (34%), about an equal proportion of ADHD-C children (35%), and smaller proportions of children with ASD (18%), ADHD-I (6%), and ASD with comorbid ADHD (6%). The “impaired” class was mainly composed of children with ASD with comorbid ADHD (45%), followed by children with ADHD-C (37%), ASD (10%), and ADHD-I (7%). There was one TD child who fell into the “impaired” class.

Figure 2b illustrates the distribution of the children between the EF classes for each diagnostic group. Most TD children were in the “above average” class (79%) with the remaining in the “average” class, except for one TD child falling into the “impaired” class.

The majority of children with ADHD were distributed between the “average” (34%) and “impaired” (63%) classes. The distribution of children with ADHD in “average” and “impaired” classes was similar across ADHD subtypes: ADHD-C in impaired class: 63%, ADHD-I in impaired class: 64%. Two children with ADHD-C were in the “above average” class.

Children with ASD were primarily in the “impaired” class (78%), with 20% in the “average” class. When comparing the distribution of children in EF classes with ASD or ASD with comorbid ADHD, there were major differences: 47% of children with ASD were in the “impaired” class, whereas 92% of children with both ASD and ADHD were in the “impaired” class.

These qualitative results were confirmed by the results of the mixture regression of diagnosis on the latent class, revealing that each EF class significantly differed in the proportion of diagnostic groups (*p*s < 0.001) (see Supplementary Table S2). Specifically, the diagnostic group with the highest probability of being in the “above average” class were TD children (probability = 0.99). The most likely diagnostic group to be in the “average” class was children with ADHD (probability = 0.55), followed by children with ASD (probability = 0.28). Children in the “impaired” class were most likely to have ASD (probability = 0.61), followed by ADHD (probability = 0.39). Although children with ASD had a 0.61 probability of being the “impaired” class, these children also had a 0.28 probability of being in the “average” class, demonstrating that some children with ASD have intact EF while others are impaired. Similarly, children with ADHD had comparable probabilities of being in the “average” and “impaired” classes (0.55 and 0.39, respectively), demonstrating that children with ADHD may have different levels of EF.

### Mixture Regression Analysis: Socioemotional variables

**Figure 3:**
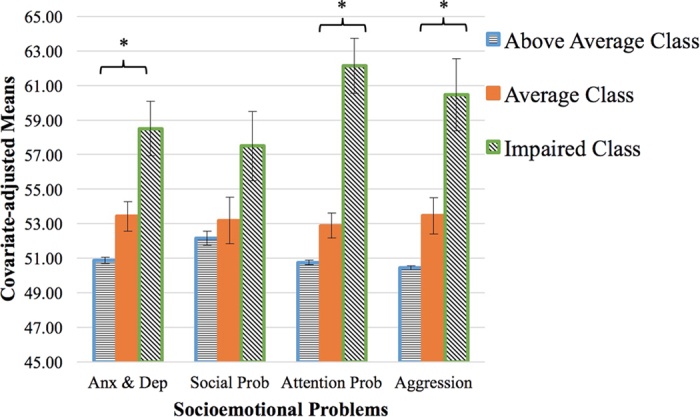
Differences in socioemotional problems between EF classes. Each class differed from each other on symptoms of anxiety/depression, attention problems, and aggression as measured by the Child Behavior Checklist after controlling for diagnosis. *Indicates a significant difference between all classes indicated by non-overlapping confidence intervals.

EF classes predicted robust phenotypic differences between children (Fig. 3, Supplementary Table S3). For every distal outcome but social problems, EF classes significantly differed from one another after adjusting for diagnosis. Children in the “above average” class had the fewest socioemotional problems, followed by the “average” class, while children in the “impaired” class had the highest level of anxiety/depression, attention problems, and aggression.

## Discussion

Using an RDoC framework^26^, this study examined EF in a mixed group of TD children and children with prevalent neurodevelopmental disorders. Latent profile analysis identified three subgroups that displayed either consistently above average EF, average EF, or clinically impaired EF. Importantly, the EF classes did not reproduce the diagnostic groups, suggesting there is heterogeneity in EF abilities within these diagnostic categories. Further, the EF classes predicted differences in a range of socioemotional problems from anxiety/depression to aggression, validating the clinical importance of the EF classes. The results of this study emphasize the presence of an “impaired” group of children, which included children with both ADHD and ASD, who might benefit from targeted EF intervention.

The EF classes did not exhibit distinct patterns of strengths and deficits in EFs, demonstrating the dimensional nature of EF abilities across children. The “average” and “impaired” EF groups included a mix of diagnoses, suggesting EF abilities do not accurately distinguish children with ASD and ADHD. In addition, the TD group did not cleanly fit into one EF category, with the majority of the TD sample falling into the “above average” class while others fell into the “average” class. These results suggest that not only is there heterogeneity in EF abilities in neurodevelopmental disorders, there is also heterogeneity (albeit less) among the TD population. Although other studies have acknowledged heterogeneity of EF within TD children^52^, many studies of clinical populations continue to employ case-control designs, which assume that both the clinical populations and controls are homogeneous in their EF abilities. Overall, the current results emphasize the importance of assessing individual differences in EF for both clinical and typical child populations.

Addressing heterogeneity within clinical populations is especially important in light of the impact of comorbidity between ASD and ADHD on EF. The results of this study highlight that this comorbid group may be experiencing a “double hit”, where impaired EF was almost guaranteed: 92% of children in the ASD + ADHD group were in the impaired EF class. In comparison, only 47% of children with ASD and 63% of children with ADHD were in the impaired class. These results emphasize the need to take ADHD and ASD symptomatology into account when assessing EF abilities in children, regardless of the child’s primary diagnosis.

Contrary to the consistency in EF subscale scores we observed, several previous studies point to distinct EF profiles in children with ADHD and ASD, such that some aspects of EF are clinically elevated while others remain relatively intact. For example, evidence suggests that there are profound impairments in inhibition in children with ADHD, but that children with ASD have higher impairments in flexibility and planning^8^,^18^,^21^. Here, we report consistent deficits across EF subdomains in children. The discrepancy between our results and past literature may be explained by methodological differences, where past studies used variable-centered approaches (group averaging). By using a person-centered approach, this study took into account the heterogeneity of EF within diagnostic groups, emphasizing individual differences in EF that are overlooked with variable-centered approaches.

The importance of studying individual differences in EF is highlighted in a previous study of EF differences in children with ASD, ADHD, and TD children^18^. Although the investigators found that children with ASD had clinically elevated scores across all EF subscales of the BRIEF compared with ADHD and TD children, only about 40% of the ASD sample exhibited elevated symptoms across all EF subscales (excluding organization of materials). Despite flexibility being the most consistently reported EF deficit in ASD, only 69% (and 66% in another study^53^) of children showed clinical impairment in flexibility. These data demonstrate that only subsets of individuals within diagnostic groups have clinically impaired EF abilities.

A positive association between EF class and various socioemotional problems validated the clinical significance of the three-class solution. This fits with previous reports of relationships between EF and other behaviors important for daily life, such as physical health, academic success, and job success in adulthood^11^. The children in the “impaired” EF class may not be solely experiencing EF problems, but also higher levels of psychological dysfunction, highlighting the need to identify these children for targeted treatment.

The existence of an “impaired” EF group with consistent dysfunction across all EF subdomains emphasizes the need to target therapies for all EFs, and not just specific subdomains. Alternatively, it is possible that an intervention for one subdomain may generalize to improvements in other EFs. Of utmost importance is identifying the children who need EF intervention. Not all children with ASD or ADHD were in the “impaired” group, but very few were in the “above average” group. This suggests that a diagnosis of ASD or ADHD may be a sign of impaired EFs, but not all these children need an EF intervention. In addition, children with ASD with comorbid ADHD were at higher risk for having impaired EF than children with only an ASD diagnosis. This corroborates findings that elevated ADHD symptoms in children with ASD exacerbate ASD symptomatology^54^. Thus, children with ASD with comorbid ADHD should be specifically screened for clinically elevated EF deficits.

EF training is effective as early as preschool-age, and the gains in EF due to intervention extend to improvements in school, including success in verbal and math skills^29^. Training has been shown to be specifically efficacious in ADHD^55^ and ASD^28^, improving not only EF, but also symptomatology, such as inattentive symptoms in ADHD and social skills in ASD. In sum, having a neurodevelopmental disorder may be a sign that a child needs an EF intervention, but does not guarantee its necessity. Instead, children with ASD and ADHD should be assessed on their current EF abilities and then offered intervention if scores indicate clinical impairment.

Here, we used a person-centered analysis to demonstrate the heterogeneity of executive functions within clinical and typically developing groups, but these findings should be interpreted in light of the following limitations. The latent profile analysis relied mainly on parent rating scales of EF, which may introduce rater bias due to the parent reporting on children’s symptoms^18^. Therefore, the interpretation of the current results is limited by measurement issues, and may not generalize to performance-based measures of EF. Future studies should seek to replicate the three-class solution found here using a breadth of performance-based measures of EF, including measures of working memory, inhibition, and flexibility. One of the performance-based EF measures used in this study, the statue subscale, was not an ideal measure of EF for the age range under investigation, which led to little variability in scores in the typically developing sample. Future research should obtain performance-based measures that are appropriate for both the age range and clinical groups studied to validate and extend the present findings. Finally, the measure of socioemotional problems in this study was a parent-report, and because the latent profile analysis relied on many parent-report scales, the relationship between the subgroups and socioemotional problems may be confounded by rater bias. To ensure the relationship between EF subgroups and children’s socioemotional problems is not confounded by rater bias, future studies should collect multiple measures of children’s functioning, such as self-, teacher-, and clinician-reported measures, to assess differences between the EF classes.

Following RDoC guidelines, this study used an exploratory approach to identify subgroups of children based on EF ability using behavioral variables (EF scores) to provide insight into mental health disorders. To extend these findings, future research should identify biological variables, such as functional brain connectivity, that differentiate the EF classes. In this way, these results may provide the foundation for discovering a unique brain-based marker for EF dysfunction, which can be used to identify children who would most benefit from EF interventions.

## Additional Information

**How to cite this article**: Dajani, D. R. *et al.* Heterogeneity of executive functions among comorbid neurodevelopmental disorders. *Sci. Rep.* **6**, 36566; doi: 10.1038/srep36566 (2016).

**Publisher’s note**: Springer Nature remains neutral with regard to jurisdictional claims in published maps and institutional affiliations.

## Supplementary Material

Supplementary Information

## Figures and Tables

**Table 1:** Descriptive statistics.

	ASD (*n* = 30)		ASD + ADHD (*n* = 66)		ADHD (*n* = 93)		TD (*n* = 128)	
	Range	Mean (SD)	Range	Mean (SD)	Range	Mean (SD)	Range	Mean (SD)
Age	8.00–12.50	9.76 (1.36)	8.00–12.92	10.45 (1.40)	8.00–12.42	9.79 (1.21)	8.00–12.58	10.03 (1.18)
Gender	M = 23 F = 7	—	M = 55 F = 11	—	M = 72 F = 21	—	M = 98 F = 30	—
VCI	57–148	106.57 (18.71)	71–148	108.39 (18.15)	85–148	111.57 (13.42)	91–148	119.17 (12.56)
PRI	79–135	112.53 (12.73)	67–127	103.99 (13.56)	82–143	109.14 (12.22)	84–147	112.35 (12.70)
WMI	56–129	103.90 (15.27)	34–135	93.57 (18.30)	74–126	102.32 (12.03)	83–148	111.78 (14.46)
PSI	68–131	92.59 (14.41)	21–133	85.92 (17.08)	73–126	94.40 (12.86)	73–144	101.69 (12.97)
FSIQ	67–132	106.10 (14.88)	63–135	99.99 (15.98)	82–136	107.31 (11.67)	88–147	115.76 (12.23)
ADOS-2 Total	7–18	12.91 (2.95)	7–22	12.24 (4.21)	—	—	—	—
ADOS-G CS	8–21	12.91 (2.95)	7–19	11.63 (3.11)	—	—	—	—
ADI A Total	9–30	20.41 (5.67)	7–29	19.57 (5.77)	—	—	—	—
ADI B Total	7–23	15.97 (4.09)	4–25	15.84 (4.81)	—	—	—	—
ADI C Total	3–10	6.21 (1.76)	2–12	6.00 (2.04)	—	—	—	—
ADHD Hyp	0–7	2.47 (2.00)	0–9	4.48 (2.44)	0–9	4.60 (2.95)	0–3	0.19 (0.54)
ADHD Inatt	0–8	3.37 (2.67)	0–9	6.73 (2.32)	2–9	6.80 (1.91)	0–3	0.16 (0.49)
CPRS-R H/I	50–78	60.67 (8.30)	45–90	70.03 (10.70)	43–90	71.38 (12.44)	41–62	47.57 (5.30)
CPRS-R Inatt	43–90	62.29 (11.59)	55–90	71.24 (8.66)	55–90	72.40 (8.13)	40–58	44.61 (4.59)
CPRS-3 H/I	52–90	66.09 (16.20)	44–90	75.59 (12.79)	45–90	72.33 (15.63)	38–64	45.38 (5.78)
CPRS-3 Inatt	51–79	66.09 (8.84)	45–90	76.38 (12.55)	58–90	74.03 (10.30)	35–63	44.70 (6.96)

Note: There are 4 individuals with ASD who have missing comorbidity information. ASD + ADHD: ASD with comorbid ADHD; VCI: verbal comprehension index; PRI: perceptual reasoning index; WMI: working memory index; PSI: processing speed index; FSIQ: full scale intelligence quotient; ADOS-2: Autism Diagnostic Observation Schedule-2; ADOS-G CS: Autism Diagnostic Observation Schedule- Generic Communication and Social Interaction; ADI: Autism Diagnostic Interview A: Reciprocal Social Interaction; ADI B: Verbal Communication; ADI C: Restricted and Repetitive Behaviors; ADHD Hyp: ADHD-Rating System, Home version, number of Hyperactivity Symptoms; ADHD Inatt: ADHD-Rating System, Home version, number of Inattention Symptoms; CPRS-R H/I: Conners Parent Rating Scales Revised long version, DSM-IV Hyperactivity/Impulsive T score; CPRS-R Inatt: DSM-IV Inattentive T score; CPRS-3 H/I: Conners 3^rd^ Edition, DSM-IV-TR Hyperactivity/Impulsive T score; CPRS-3 Inatt: DSM-IV-TR Inattentive T score.

**Table 2:** Latent Profile Analysis results.

	1 class	2 classes	3 classes	4 classes	5 classes	6 classes
LL	−11512.94	−10630.78	−10464.54	−10377.23	−10328.88	−10284.36
AIC	23065.88	21323.57	21013.08	20860.45	20785.76	20718.72
BIC	23141.31	21440.48	21171.48	21060.34	21027.14	21001.57
SA BIC	23077.87	21342.15	21038.26	20892.23	20824.14	20763.69
Entropy	—	0.92	0.88	0.87	0.85	0.86
LMR	—	2 *v* 1	3 *v* 2	4 *v* 3	5 *v* 4	6 *v* 5
	—	1736.95^a^	327.33^b^	171.92^c^	95.19^d^	87.67^c^
N for each class	N = 321	C1 = 136	C1 = 105	C1 = 97	C1 = 63	C1 = 68
		C2 = 185	C2 = 78	C2 = 130	C2 = 65	C2 = 62
			C3 = 138	C3 = 60	C3 = 53	C3 = 53
				C4 = 34	C4 = 111	C4 = 17
					C5 = 29	C5 = 92
						C6 = 29

Note: One hundred different sets of start values were generated and a full iteration was completed for the ten best sets. LL = Log Likelihood; AIC* *=* *Aikake Information Criterion, BIC* *=* *Bayesian Information Criterion; SA BIC* *=* *Sample Adjusted BIC; LMR* *=* *Lo, Mendell, Rubin Likelihood Ratio Test; ^a^*p *< 0.001, ^b^*p *=* *0.07, ^c^*p *>* *0.10, ^d^*p *=* *0.02.
