# Supplementary material for: Heterogeneity of executive functions among comorbid neurodevelopmental disorders
Source: Sci Rep. 2016 Nov 9;6:36566. doi: 10.1038/srep36566 (PMC5101520; doi:10.1038/srep36566)
Supplement: Supplementary Information [file srep36566-s1.pdf]

**Heterogeneity of executive functions among comorbid neurodevelopmental disorders**

Dina R. Dajani[1], Maria M. Llabre[1], Mary Beth Nebel[2,3],
Stewart H. Mostofsky[2,3,4] & Lucina Q. Uddin[1,5]

[1]Department of Psychology, University of Miami, Coral Gables, FL
[2]Center for Neurodevelopmental and Imaging Research, Kennedy Krieger Institute
[3]Department of Neurology, Johns Hopkins University School of Medicine
[4]Department of Psychiatry and Behavioral Sciences, Johns Hopkins University School of Medicine
[5]Neuroscience Program, University of Miami Miller School of Medicine, Miami, FL

**Supplemental Information**

Diagnostic Measures

***ADI-R.*** The Autism Diagnostic Interview- Revised (ADI-R, Rutter, Le Couteur, Lord, & Faggioli, 2005) is a parent clinical interview that assesses key diagnostic features of autism like reciprocal social interaction, communication, and repetitive/stereotyped behaviors. All participants with ASD had scores of 7 or higher on the three subscales.

***ADOS-G and ADOS-2.*** The Autism Diagnostic Observation Schedule-Generic (ADOS-G (Lord et al., 2000) and the Autism Diagnostic Observation Schedule-2 (ADOS-2, Lord et al., 2012) are child clinical interviews with structured and semi-structured parts that focus on social and communicative behaviors in children. Participants recruited prior to the release of the ADOS-2 received the ADOS-G. Community diagnoses of ASD were confirmed by a score of ≥ 7 for the total score on the ADOS-2 or the communication and social interaction score on the ADOS-G.

***Conners' PRS.*** The Conners' Parent Rating Scales- Revised, Long Version (CPRS-R:L, Conners, 1997) and the Conner's Parent Rating Scales- 3$^{rd}$ Edition, Full-length (CPRS-3, Conners, 2008) are parent reports of their child's ADHD symptoms, oppositional defiant disorder and conduct disorder. T-Scores of 60 or higher on the *DSM-IV* (for the Revised version) and *DSM-IV-TR* (for the 3$^{rd}$ Edition) Hyperactive/Impulsive or Inattentive scales were used to confirm community ADHD diagnoses. TD participants who had T-Scores >65 on either the Hyperactive/Impulsive or Inattentive scales were excluded from the study.

***ADHD-RS-IV, Home version.*** The ADHD Rating Scale-IV, Home version (DuPaul, Power, Anastopoulos, & Reid, 1998) is a parent report of their child's ADHD symptoms

over the previous 6 months. Community diagnoses of ADHD were confirmed if children met for 6 out of 9 *DSM-IV* symptoms on either the Hyperactive/Impulsive or Inattention scales. TD participants who met on 4 out of 9 symptoms on either the Hyperactive/Impulsive or Inattention scales were excluded from the study.

***DICA-IV.*** The Diagnostic Interview for Children and Adolescents IV (Reich, Welner, & Herjanic, 1997) is a structured parent interview to evaluate current psychiatric diagnoses in the child. The Attention Deficit Disorder subscale was used to: 1) confirm community ADHD diagnoses and 2) to determine whether the children with community ASD diagnoses also had comorbid ADHD. Any child who met criteria for conduct disorder, mood disorder, generalized anxiety disorder, or obsessive-compulsive disorder was excluded from the study. TD children who met criteria for any disorder were excluded. Scores on the DICA-IV and clinical judgment by a child neurologist (S.H.M) determined ADHD subtype.

Supplemental Tables

**Table S1.** Differences in indicators across classes using 10 indicators.

| | Above Average | Average | Impaired | F | Post-hoc |
|---|---|---|---|---|---|
| | Mean (SE) | Mean (SE) | Mean (SE) | | |
| BRIEF- Parent | | | | | |
| -Inhibition T | 42.27 (.71) | 55.58 (2.15) | 67.56 (1.41) | 231.51 | I > A > AA |
| -Shift T | 41.85 (.67) | 52.86 (1.97) | 67.00 (1.81) | 178.09 | I > A > AA |
| -Emotional Control T | 41.21 (.66) | 51.92 (1.67) | 62.45 (1.51) | 146.67 | I > A > AA |
| -Initiation T | 42.63 (.91) | 54.41 (1.69) | 65.97 (1.11) | 287.39 | I > A > AA |
| -Working Memory T | 42.02 (1.05) | 57.86 (2.81) | 71.58 (.83) | 557.59 | I > A > AA |
| -Plan/Organize T | 41.43 (.91) | 55.76 (2.74) | 70.00 (.86) | 582.5 | I > A > AA |
| -Org Materials T | 44.96 (1.22) | 56.34 (1.44) | 64.61 (.83) | 178.22 | I > A > AA |
| -Monitor T | 39.60 (1.20) | 56.16 (2.17) | 68.23 (1.05) | 428.43 | I > A > AA |
| WISC B-Digit span | 12.11 (.33) | 11.43 (.48) | 10.09 (.30) | 16.71 | AA=A>I |
| Statue Total | 27.25 (.41) | 24.10 (1.09) | 20.31 (.89) | 30.16 | AA>A>I |

All F-tests were significant at the $p < .001$ level. Note that for the BRIEF, higher scores indicate greater impairment, but for the B-Digit span and Statue, higher scores indicate lower impairment. I: Impaired class; A: Average class; AA: Above Average class; Org Materials T: Organization of Materials T-score; WISC B-Digit span: Wechsler Intelligence Quotient for Children IV Backward Digit span scaled score; Statue Total: total score on the NEPSY-II statue subtest.

**Table S2.** Logistic regression of diagnosis on latent class.

| | Probability (SE) | Odds Ratio (SE) | 95% CI | $\chi^2$ |
|---|---|---|---|---|
| Above Average | | | | AA *v* I |
| TD | 0.99 (.01) | 118.36 (123.46) | 15.32-914.32 | 5502.96[c] |
| ADHD | 0 (0) | --[a] | --[a] | |
| ASD | .01 (.01) | --[b] | --[b] | |
| Average | | | | A *v* AA |
| TD | 0.18 (.08) | --[b] | --[b] | 123.44[c] |
| ADHD | 0.55 (.08) | --[b] | --[b] | |
| ASD | 0.28 (.06) | --[b] | --[b] | |
| Impaired | | | | |
| TD | 0 (0) | --[a] | --[a] | I *v* A |
| ADHD | 0.39 (.05) | .33 (.13) | .15-.72 | 17.92[c] |
| ASD | 0.61 (.05) | --[b] | --[b] | |

Note: Uninterpretable odds ratios for: [a] = 0 (CI: 0-0) and [b] = 1 (CI: 1-1). [C]= $p < .001$

**Table S3.** Differences in behavioral problems between EF classes

| | Intercept | SE | 95% CI |
|---|---|---|---|
| **Distal Outcome** | | | |
| 1. Anxiety/Depression | | | |
| Above average | 50.87 | 0.18 | 50.51-51.23 |
| Average | 53.42 | 0.87 | 51.72-55.12 |
| Impaired | 58.51 | 1.57 | 55.43-61.58 |
| 2. Social Problems | | | |
| Above average | 52.16 | 0.40 | 51.38-52.94 |
| Average | 53.19 | 1.36 | 50.52-55.85 |
| Impaired | 57.49 | 2.00 | 53.57-61.42 |
| 3. Attention Problems | | | |
| Above average | 50.75 | 0.13 | 50.49-51.01 |
| Average | 52.88 | 0.73 | 51.46-54.30 |
| Impaired | 62.15 | 1.60 | 59.02-65.28 |
| 4. Aggression | | | |
| Above average | 50.44 | 0.12 | 50.20-50.68 |
| Average | 53.46 | 1.05 | 51.40-55.51 |
| Impaired | 60.47 | 2.08 | 56.39-64.54 |

Four mixture regression analyses were performed, one for each distal outcome. Intercepts are the means of the distal outcome adjusted for diagnosis. Non-overlapping confidence intervals indicate significant differences between EF classes. CI: confidence interval.

**Results of supplementary latent profile analysis excluding NEPSY-II Statue subtest**

**Table S4.** Latent Profile Analysis results excluding NEPSY Statue.

| | 1 class | 2 classes | 3 classes | 4 classes | 5 classes | 6 classes |
|---|---|---|---|---|---|---|
| LL | -10554.78 | -9697.28 | -9532.18 | -9446.06 | -9398.30 | -9357.48 |
| AIC | 21145.56 | 19450.55 | 19140.37 | 18988.12 | 18912.61 | 18850.96 |
| BIC | 21213.39 | 19556.06 | 19283.56 | 19168.96 | 19131.17 | 19107.21 |
| SA BIC | 21156.30 | 19467.25 | 19163.03 | 19016.75 | 18947.21 | 18891.53 |
| Entropy | - | 0.92 | 0.88 | 0.88 | 0.86 | 0.86 |
| | - | 2 v 1 | 3 v 2 | 4 v 3 | 5 v 4 | 6 v 5 |
| LMR LRT | - | 1685.79[a] | 324.56[a] | 169.32 | 93.88[a] | 102.75[a] |
| N for each class | N=320 | C1=136 | C1=102 | C1=59 | C1=64 | C1=62 |
| | | C2=184 | C2=80 | C2=129 | C2=56 | C2=63 |
| | | | C3=138 | C3=98 | C3=63 | C3=14 |
| | | | | C4=34 | C4=29 | C4=56 |
| | | | | | C5=108 | C5=96 |
| | | | | | | C6=29 |

Note: One hundred different sets of start values were generated and a full iteration was completed for the ten best sets. LL= Log Likelihood; AIC= Aikake Information Criterion, BIC= Bayesian Information Criterion; SA BIC= Sample Adjusted BIC; LMR= Lo, Mendell, Rubin Likelihood Ratio Test; BLRT= Bootstrapped Likelihood Ratio Test. [a]$p < .05$.

**Table S5.** Differences in indicators across classes excluding NEPSY Statue.

| | Above Average | Average | Impaired | F | Post-hoc |
|---|---|---|---|---|---|
| | Mean (SE) | Mean (SE) | Mean (SE) | | |
| BRIEF- Parent | | | | | |
| -Inhibition T | 42.1 (0.49) | 55.33 (0.97) | 67.51 (0.97) | 231.51 | I > A > AA |
| -Shift T | 41.65 (0.54) | 52.88 (1.16) | 66.83 (1.07) | 182.36 | I > A > AA |
| -Emotional Control T | 41.02 (0.51) | 51.86 (1.06) | 62.35 (0.99) | 151.22 | I > A > AA |
| -Initiation T | 42.5 (0.56) | 53.93 (0.72) | 66.06 (0.78) | 291.66 | I > A > AA |
| -Working Memory T | 41.79 (0.54) | 57.26 (0.89) | 71.74 (0.59) | 566.83 | I > A > AA |
| -Plan/Organize T | 41.21 (0.51) | 55.5 (0.78) | 69.95 (0.62) | 555.09 | I > A > AA |
| -Org Materials T | 44.72 (0.74) | 56.13 (0.97) | 64.64 (0.64) | 185.65 | I > A > AA |
| -Monitor T | 39.33 (0.67) | 55.8 (0.91) | 68.25 (0.66) | 423.69 | I > A > AA |
| WISC B-Digit span | 12.3 (0.31) | 11.51 (0.36) | 9.97 (0.27) | 16.70 | AA=A>I |

All F-tests were signficant at the $p < .001$ level. Note that for the BRIEF scores higher indicates greater impairment, but for the WISC, higher scores indicate less impairment. I: impaired class; A: average class; AA: above average class; PlanOrg T: Org Materials: Organization of Materials T-score; WISC B-Digit span: Wechsler Intelligence Quotient for Children IV Backward Digit span scaled score.

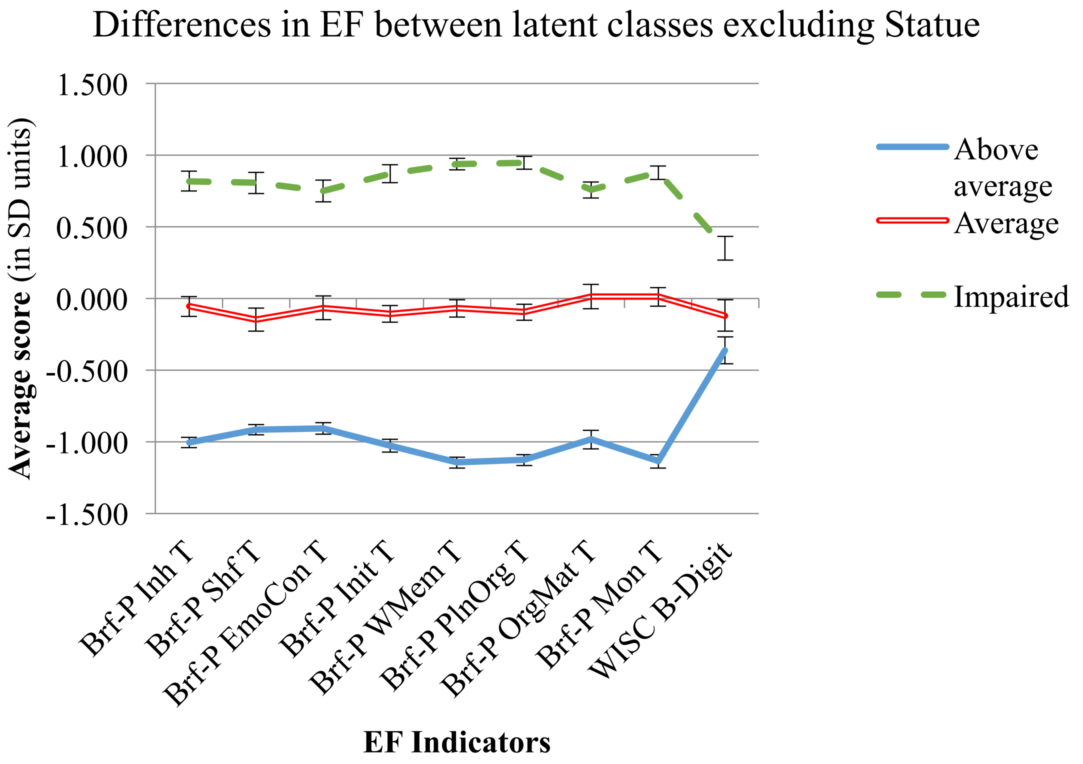


**Figure S1.** Latent Profile Analysis results excluding NEPSY Statue subtest. The three EF classes differed on their scores across the indicators. Scores are in standard deviation units based on data from the present sample. The WISC Backward Digit span was reverse-scored such that for all indicators, higher scores indicate greater impairment. Note: All EF indicators had a significant difference between Above average, Average, and Impaired groups except the WISC B-Digit, which only had a difference between Above average/Impaired groups and Average/Impaired groups. Brf-P Inh T: BRIEF-Parent report inhibition T-score; Shf T- Shift T-score; EmoConT: Emotional Control T-score; Init T: Initiation T-score; WMem T: Working memory T-score; PlnOrg T: Plan/Organize T-score; OrgMat: Organization of Materials T-score; Mon T: Monitor T-score; WISC B-Digit: Wechsler Intelligence Quotient for Children IV Backward Digit span scaled score (reverse scored); Statue Total: total score on the NEPSY-II statue subtest (reverse scored).

Conners, C. K. (1997). *Conners' Rating Scales--revised: User's Manual*: Multi-Health Systems, Incorporated.

Conners, C. K. (2008). *Conners 3rd edition*: Multi-Health Systems Toronto, Ontario, Canada.

DuPaul, G. J., Power, T. J., Anastopoulos, A. D., & Reid, R. (1998). *ADHD Rating Scale-IV: Checklists, norms, and clinical interpretation* (Vol. 25): Guilford Press New York.

Lord, C., Risi, S., Lambrecht, L., Cook Jr, E. H., Leventhal, B. L., DiLavore, P. C., et al. (2000). The Autism Diagnostic Observation Schedule—Generic: A standard measure of social and communication deficits associated with the spectrum of autism. *Journal of autism and developmental disorders, 30*(3), 205-223.

Lord, C., Rutter, M., DiLavore, P. C., Risi, S., Gotham, K., & Bishop, S. L. (2012). *Autism diagnostic observation schedule: ADOS-2*: Western Psychological Services Los Angeles, CA.

Reich, W., Welner, Z., & Herjanic, B. (1997). Diagnostic interview for children and adolescents-IV (DICA-IV) Multi-Health Systems. *Toronto, Canada*.

Rutter, M., Le Couteur, A., Lord, C., & Faggioli, R. (2005). *ADI-R: Autism diagnostic interview--revised: Manual*: OS, Organizzazioni speciali.
